# Beyond the Surface: Mechanical and Porosity Gradients in Eroded Enamel

**DOI:** 10.1177/00220345251385464

**Published:** 2025-10-22

**Authors:** H.L. Ooi, D. Bartlett, A. Almansour, A. LeBlanc, D. White, A. Morrell, O. Addison

**Affiliations:** 1 Faculty of Dentistry, Oral and Craniofacial Sciences, King’s College London, London, UK; 2 London Metallomics Facility, King’s College London, London, UK

**Keywords:** erosion, subsurface, porosity, demineralization, synchrotron, remineralization

## Abstract

Enamel erosion alters the structural integrity of the tooth surface, which can be measured using indentation techniques. However, traditional single-load indentation methods assume homogeneity within the eroded enamel, overlooking potential stratification within the subsurface lesion. This study investigates the presence of mechanical and porosity gradients within the enamel following simulated dietary acid exposure and examines how lesion depth and structure change with continued erosion. We applied varying-load micro-indentation to human enamel subjected to citric acid challenge, revealing a distinct stratification of mechanical properties. A soft superficial layer (~1- to 2-µm thick) exhibited significantly reduced hardness and was easily removed by ultrasonication, indicating its fragility. Beneath this layer, mechanical properties stabilized despite prolonged acid exposure (~3 min), suggesting a saturation point in lesion development. Profilometric analysis confirmed that although material loss increased with erosion time, the depth of the altered subsurface zone remained constant. To explore the porosity distribution, we used a novel gold nanoparticle labeling technique coupled with synchrotron-based X-ray fluorescence imaging. Nanoparticles (~20 nm) penetrated to depths of 15 to 20 µm, aligning closely with mechanical gradients inferred from indentation measurements. These findings indicate that subsurface enamel exhibits not only mechanical stratification but also corresponding variations in porosity. Our results demonstrate the limitations of single-load indentation in characterizing erosion-affected enamel and highlight the utility of multiload approaches in detecting structural heterogeneity. The correlation between mechanical softening and increased porosity suggests that the enamel subsurfaces are differentially affected. These findings raise important implications for therapeutic intervention: should remineralization strategies shift from bulk mineral delivery to layer-specific, functionally informed repair?

## Graphical abstract

**Graphical Abstract:**
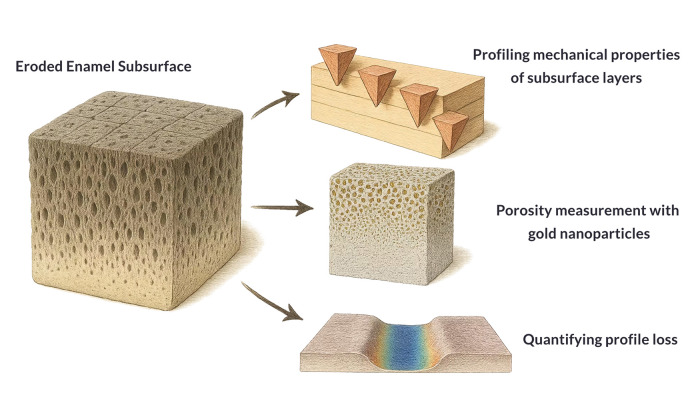

## Introduction

The global market for products preventing early dental and gingival diseases exceeds $55 billion (Statista 2025), with enamel-preserving products forming a major segment. These typically modify enamel solubility via fluoride or promote remineralization (Young et al. 2006; Sulyanto et al. 2021; Mazzoleni et al. 2023). However, the ability to maintain or restore enamel’s hierarchical structure and mechanical integrity is limited by insufficient understanding of the subsurface layer’s porosity, hindering efforts to optimize infiltration and remineralization.

Initial acid exposure weakens the subsurface without immediate surface loss (Larsen 1990), but continued exposure leads to measurable tissue removal (Bartlett 2016), influenced by structural factors such as hydroxyapatite crystallite orientation and ionic substitutions (Jones et al. 2013; Ooi et al. 2025). In vitro studies often quantify erosion using 3-dimensional (3D) profilometry to measure “step heights” (Austin et al. 2015; Mylonas et al. 2018; Mylonas et al. 2019; Almansour, Bartlett, et al. 2024). While this quantifies material loss, understanding the condition of the remaining enamel is critical for remineralization and prevention strategies. In sound enamel, mineral comprises ~85% to 87% by volume (95%–96% by weight), water ~12% by volume (3%–4% by weight), and organic matter ~1% to 2% by volume (0.5%–1% by weight) (Angmar et al. 1963; Robinson et al. 1971). This small fraction of water and organic components creates a nanoporous network that facilitates diffusion into enamel and its response to chemical challenges. However, beyond surface observations, little is known about how this network evolves after dietary acid exposure or its penetrability in a physiologically hydrated state.

Current understanding of eroded enamel is based on electron microscopy and surface imaging techniques, which have provided valuable insight into the structure of affected tissue. These approaches have helped establish the presence of a demineralized subsurface layer approximately 2- to 12-µm thick, based on semi-quantitative methods such as scanning electron microscopy (9–12 µm) and profilometry following ultrasonication (2–5 µm) (Eisenburger et al. 2000; Eisenburger et al. 2004). Despite careful sample handling, including freeze drying to preserve surface integrity, these remain surface-level analyses and require sectioned samples. As such, they offer limited information on internal, layer-specific mechanical and porosity gradients or on the temporal progression of lesions.

Earlier porosity studies by Poole et al. (1961) estimated pore size by infiltrating alcohols of varying refractive indices and measuring the resulting birefringence changes. This approach provides useful insight, although interpretation is complicated by the enamel’s anisotropic structure. The sensitivity of birefringence to the orientation of primary and secondary crystallites makes it difficult to distinguish pore-related changes from orientation effects (Schmidt and Keil 1971). In addition, the method relied on destructive sampling of ground, dehydrated sections, limiting its suitability for repeated or 3D analysis under hydrated, physiologically relevant conditions.

Current research often also overlooks mechanical gradients in the eroded enamel subsurface. Micro-indentation is commonly used to assess the mechanical properties of eroded enamel (Hannig et al. 2008; Zheng et al. 2009; An et al. 2012; Chew et al. 2014). These tests typically use single loads (often 0.05 or 0.1 kgF), which can detect softening but are too large to isolate an extremely weak superficial layer. As a result, values often reflect only part of the partially demineralized region, leaving both the outermost and deeper zones uncharacterized. While nanoindentation provides high spatial resolution for probing individual crystallites, its limited penetration prevents full assessment of erosive lesions, which usually span 10 to 20 µm (Cuy et al. 2002; He and Swain 2007; Cheng et al. 2009). In addition, studies using sectioned enamel may not reflect natural crystal orientation in vivo, a known determinant of the mechanical response (Cuy et al. 2002; Xie et al. 2009). Almansour, Addison, et al. (2024) varied indentation loads and analyzed deviations from the indentation size effect to assess the occlusal and buccal microstructure after 60 min of erosion. However, subsurface layer thickness was not estimated, and the prolonged erosion exceeds typical short-term exposures (1–3 min), leaving lesion progression over clinically relevant time scales unclear.

To fully characterize early erosion, complementary methods are needed that combine short-term mechanical testing with imaging capable of resolving subsurface porosity, which remains challenging, even with high-resolution imaging. Techniques such as focused ion beam scanning electron microscopy (SEM) and synchrotron-based ptychography offer nanometer-level resolution but require ultrathin sections, which can introduce artifacts and risk separating prism sheaths (Besnard, Marie, Buček, et al. 2022; Besnard, Marie, Sasidharan, et al. 2022). Thicker sections (~2.4 µm) reduce some preparation issues but are insufficient to capture erosive lesions extending 10 to 20 µm. Even less destructive synchrotron microtomography, with ~300-nm resolution, may still overlook small pores within the hydroxyapatite matrix (Besnard et al. 2021).

Other laboratory techniques, such as optical coherence tomography, detect porosity via light backscatter but have limited resolution (12–25 µm), insufficient to capture nanoscale surface variation (Wilder-Smith et al. 2009). Micro-radiography, commonly used to assess enamel mineral content, relies on densitometric readings to indirectly infer pore presence. However, it cannot reliably distinguish between empty spaces and organic material. Reported lateral sampling ranges from 10 to 100 µm (Angmar et al. 1963; Hall et al. 1997). Given that Eisenburger et al. (2004) estimated the demineralized subsurface layer to be only 9- to 12-µm thick, there remains no direct quantification of pore size or porosity gradients in enamel, correlated with mechanical changes, following dietary acid exposure.

The overarching aim of this study is therefore to provide a comprehensive understanding of the demineralized subsurface enamel layer outlined in Figure 1. First, we seek to discriminate the response to indentation associated with depth of penetration into the subsurface lesion. Second, we aim to assess the temporal evolution of any functionally graded zones and how they evolve over progressive erosion time. Finally, we aim to quantify enamel subsurface porosity in terms of variation of gap sizes and the depth of impact following enamel erosion using a novel labeling approach.

**Figure 1.**
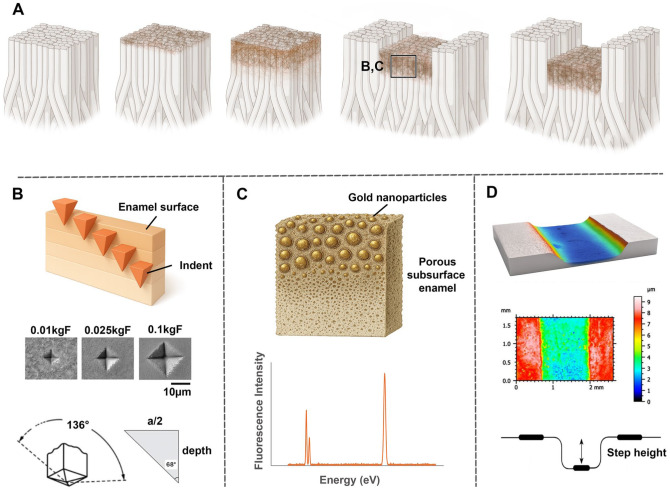
Overview of techniques used to characterize the eroded enamel subsurface. (**A**) Schematic illustrating the progression of enamel erosion over time, showing the evolution of a subsurface demineralized layer that deepens and eventually leads to surface material loss. (**B**) Micro-indentation at varying loads enables probing of different depths within the enamel subsurface. Representative indentations and schematic geometry of the Vickers indenter are shown, including the method for calculating indentation depth (Appendix Fig. 1). (**C**) Visualization of gold-coated magnetic nanoparticles penetrating enamel porosities within the eroded subsurface. The lower panel shows a representative synchrotron-based X-ray fluorescence spectrum used to detect gold at the L-edge. (**D**) Three- and two-dimensional profilometry images illustrating a “step height” at the eroded lesion site, flanked by 2 protected (noneroded) reference zones with a schematic representation of the step height measurement principle. Images in B, C, and D were created with Illustrae.co, and image A was created with BioRender.com.

## Materials and Methods

### Tooth Sample Preparation

Thirty-two enamel samples were prepared from the midbuccal/palatal surfaces of caries-free human molars (REC16/SW/0220). Teeth were disinfected in 5% sodium hypochlorite (3 d), stored in deionized water, and sectioned with a water-cooled diamond saw (XL12205, Benetec). Halved crowns exposing 2 facial surfaces were mounted in epoxy resin (EpoThin 2, Buehler) under vacuum. After curing, surfaces were polished with 500- to 4,000-grit silicon–carbide discs using a Struers LaboForce-30 (10 N, 150 RPM). Four samples were further thinned to 5 mm for nanoparticle exposure. All samples were ultrasonicated (GP-70, Nusonics) in distilled water (pH 7) for 15 min. A 1- × 3-mm enamel window was exposed using PVC tape with 2 reference zones.

### Erosive Challenge

A 0.3% citric acid solution (pH 2.7) was prepared by dissolving 3 g anhydrous citric acid in 1,000 mL distilled water and titrated with 0.1 M NaOH. pH was confirmed with a calibrated meter (Mettler Toledo).

Twenty-four samples underwent a single erosion cycle of 1, 10, or 60 min (*n* = 8/group). An additional 8 samples underwent progressive erosion at 20 s, 40 s, 1 min, 2 min, 3 min, 4 min, 5 min, 10 min, 20 min, 40 min, and 60 min. Samples were immersed in 30 mL acid and agitated at 62 rpm (Stuart SSM1, Bibby Scientific), then rinsed and air dried overnight. Samples for nanoparticle labeling were eroded for 10 min before tape removal and inspection.

### SEM

Samples eroded for 1, 10, and 60 min were imaged surface up and in cross section using a Jeol JCM7000 (Jeol Ltd) in secondary electron imaging mode at 10 kV with carbon coating (10 nm).

### Surface Microhardness Testing and Depth Gauging of Subsurface Eroded Layers

A Vickers microhardness tester (DuraScan 20G5, Emco-Test GmbH) with a 15-s dwell time was used. For the 24 single-cycle samples, 10 loads were applied: 0.01, 0.02, 0.025, 0.05, 0.1, 0.2, 0.3, 0.5, 1, and 2 kgF. Testing was repeated after 30-min ultrasonication. For the 8 additional samples, selected loads (0.01, 0.02, 0.1, and 0.5 kgF) were used on control and following each erosion time point (20 s, 40 s, 1 min, 2 min, 3 min, 4 min, 5 min, 10 min, 20 min, 40 min, and 60 min).

Each measurement was the average of 3 indents, spaced 2.5× the diagonal length, per ASTM E384 (2017). Indents were imaged using a digital microscope (Keyence VHX-S750E) at ×700 to ×2,500. The Vicker’s hardness number (VHN) was calculated as

$$VHN=\frac{1.854xF}{D^{2}}$$

where *F* is the load (kgF) and *D* is the average diagonal length (mm). The indentation depth was estimated based on the Vickers indenter geometry (Fig. 1B and Appendix Methods).

### Surface Profilometry and 3D Step Height Measurements

Lesion depth was measured with a noncontact white-light profilometer (P3300, TaiCaan) using a 12-µm spot, 3,000-µm gauge range, and 0.01-µm z-resolution. The 501 × 501 scan grid used a 10-µm step at 1,000 Hz. Step height was calculated using MountainsMap® software (Digitalsurf).

### Nanoparticle Labeling of Eroded Surfaces

The eroded enamel surfaces were labeled with monodispersed gold-coated magnetic nanoparticles (20, 40, 100, 250 nm; Nanopartz), 1 size per sample (Appendix Table 1). Each suspension was ultrasonicated for 5 min, and 10 µL was applied. A magnet (11-kg pull, 133.6 mT) was placed beneath the samples for 60 min; at 30 min, 10 µL of deionized water was added to ensure constant hydration. After incubation, samples were rinsed, air-dried, reembedded in resin, and cross-sectioned with a diamond saw.

### Quantification of the Gold Label

Gold nanoparticle distribution was quantified via X-ray fluorescence (XRF) at beamline I18 (Diamond Light Source), using a 12.5-keV monochromatic source with a 2- × 2-µm beam spot and 40-ms acquisition with an attenuation depth of ~90 µm into the enamel surface (Appendix Methods). One sample was analyzed for each particle size, with mapping performed over 15 pixels in X and 150 pixels in Y (a total of 2,250 data points) for the horizontal maps and 12 pixels in X and 30 pixels in Y (a total of 360 data points) for the vertical maps. Scans were performed on cross-sectioned samples approximately 2-mm thick, extending from the enamel surface to ~50 µm into the enamel toward the enamel dentine junction (EDJ). The mean gold intensity (L-III emission detected at 11.9 keV) was calculated and normalized for intersize comparison.

### Statistical Analyses

Data were collected and recorded in Excel (2020, Microsoft Corp) and Data Analysis WorkbeNch (DAWN version 2.32.0,2023). Statistical analysis and graphing used GraphPad Prism (v10.0.0, macOS) and Python (Spyder 5.4.3). Normality was assessed using the Shapiro–Wilk test. Means and standard deviations were calculated. One-way analysis of variance (ANOVA; profilometry data), repeated measures 2-way ANOVA (progressive erosion), and 2-way ANOVA (single time point erosion) were used to assess group differences, followed by Tukey’s honestly significant difference for post hoc comparisons (α = 0.05). Further details on sample size and effect size are provided in the Appendix Methods and Appendix Table 2)

## Results

### Evolution of the Eroded Subsurface Layers with Erosion Time

The effect of erosion time on the surface indentation behavior of enamel was evaluated using 4 microhardness loads (0.01, 0.02, 0.1, and 0.5 kgF). Across all loads, hardness values decreased with increasing erosion time, followed by a plateau (Fig. 2B, D). The most pronounced decline occurred within the first minute of acid challenge, particularly at the lighter loads (0.01 and 0.02 kgF). At early erosion time points, lighter loads produced higher VHN values compared with increased loads. However, beyond 1 min, this trend reversed, with heavier loads consistently yielding higher hardness values. By 3 min, hardness values stabilized across all loads, with no statistically significant differences observed between subsequent time points. Indentation depth calculations further supported this stabilization, revealing statistically distinct penetration (*P* < 0.05) depths for each load after 3 min of erosion: 1.27 ± 0.21 µm (0.01 kgF), 1.67 ± 0.21 µm (0.02 kgF), 3.44 ± 0.17 µm (0.1 kgF), and 7.47 ± 0.11 µm (0.5 kgF), shown in Figure 2C.

**Figure 2.**
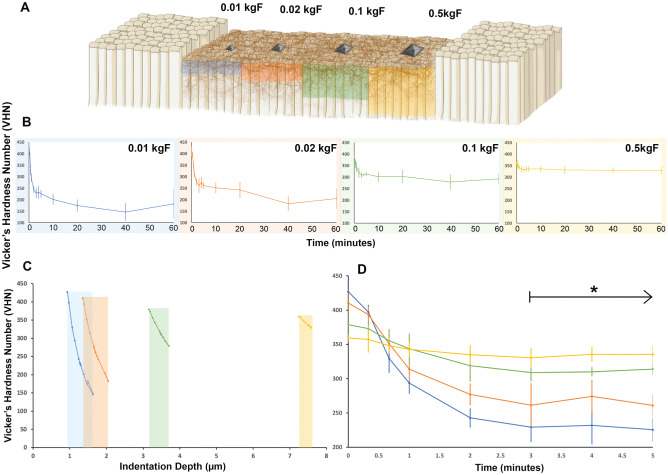
Load-dependent mechanical response of the enamel subsurface during progressive erosion. (**A**) Schematic illustration showing the conceptual basis for applying increasing indentation loads (0.01 kgF to 0.5 kgF) to probe progressively deeper regions of the eroded enamel subsurface highlighted by the respective colors. (**B**) Vickers microhardness values measured at 12 erosion time points (0 s, 20 s, 40 s, 1 min, 2 min, 3 min, 4 min, 5 min, 10 min, 20 min, 40 min, and 60 min) for each load. From left to right, the panels represent data for 0.01 kgF, 0.02 kgF, 0.1 kgF, and 0.5 kgF, respectively. (**C**) Estimated indentation depth for each load at the same erosion time points, illustrating the depth-resolved mechanical response of the lesion. (**D**) Enlarged view of microhardness trends from (B), showing a crossover near 1 min—reflecting the loss of prism elasticity due to early organic matrix disruption. Beyond 3 min, hardness values stabilize and are no longer statistically different between time points (represented by *).

Cross-sectional SEM imaging at 10 and 60 min confirmed the presence of a defined affected zone, extending to similar depths (~15–20 µm) at both time points (Fig. 3A, B). Surface-view SEM images revealed a loss of prismatic structure, which was more pronounced at 60 min than at 10 min (Fig. 3D, E). However, this did not correspond to increased lesion depth in cross section. In contrast, the 1-min exposed samples retained well-defined enamel prism patterns and polishing scratches in surface views, with minimal structural damage observed in cross section (Fig. 3C, F). While subsurface hardness remained relatively stable after 3 min, profilometry revealed a continued increase in material loss (step height) with prolonged erosion. An overall trend of increasing step height was observed up to 60 min. Notably, higher-than-expected step heights were recorded at 40 s (4.47 ± 1.30 µm) and 1 min (4.46 ± 1.71 µm), deviating from a linear progression (Fig. 4, Appendix Table 3).

**Figure 3.**
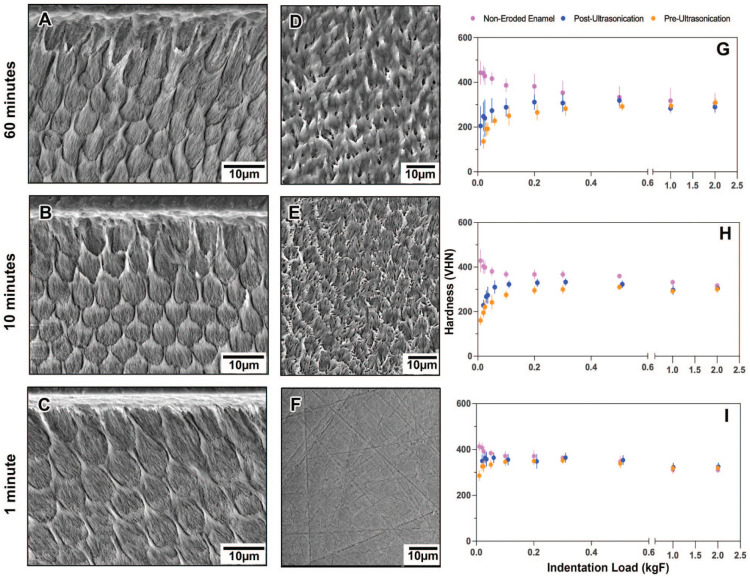
Structural and mechanical characterization of the eroded enamel surface and subsurface. (**A–C**) Scanning electron microscopy (SEM) cross-sectional images of enamel eroded for 60, 10, and 1 min, respectively. Panels A and B show similar lesion depths (~15–20 µm), whereas panel C shows minimal subsurface damage. (**D–F**) SEM surface images for the same time points. The 60-min sample (D) shows loss of prismatic structure, the 10-min sample (E) retains partially visible prisms, and the 1-min surface (F) preserves the original polished finish. (**G–I**) Vickers hardness number (VHN) plotted across 10 indentation loads (0.01–2 kgF) for uneroded control, posterosion (preultrasonication), and postultrasonication surfaces. A recovery in hardness at lower loads is observed after ultrasonication across all erosion time points.

**Figure 4.**
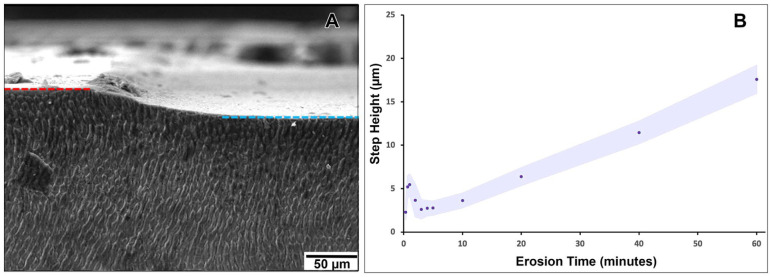
Profile loss analysis of enamel erosion. (**A**) Scanning electron microscopy image of an eroded enamel sample. The red-shaded area represents the protected region used as a reference point, while the blue-shaded area corresponds to the eroded lesion used for step height measurement. (**B**) Graph showing the increase in step height (profile loss) as a function of erosion time.

### Presence of a Superficial Weak Subsurface Layer

Surface microhardness was measured at 3 erosion time points (1, 10, and 60 min), both before and after ultrasonication. Control (noneroded) enamel samples consistently showed high hardness values at very low loads (0.01, 0.02, and 0.025 kgF), which plateaued at higher loads. In contrast, eroded samples exhibited the opposite pattern with very low hardness values at light loads that gradually increased and plateaued with heavier loads (Fig. 3G-I).

Following ultrasonication, a partial recovery in hardness was observed at light loads in the eroded groups, suggesting that ultrasonication removed a mechanically compromised outer layer. Overall, microhardness values postultrasonication were increased compared with preultrasonication measurements. The hardness trends for the 10- and 60-min erosion groups closely aligned across all loads, whereas the 1-min erosion profile more closely resembled that of the noneroded control.

Profilometry step height measurements confirmed the physical loss of material from the enamel surface after ultrasonication. The 10- and 60-min groups showed increased step heights of 1.27 ± 0.7 µm and 1.45 ± 0.76 µm, respectively. Conversely, the 1-min group exhibited a reduction in step height (−1.41 ± 1.44 µm), indicating a slight profile gain. This aligns with the nonlinear behavior observed in enamel samples undergoing progressive erosion at the 40-s and 1-min time points.

### Porosity Distribution within the Subsurface Enamel

The eroded enamel surface displays a modified subsurface structure relative to the deeper layers of sound enamel, with SEM images revealing the presence of small pores within the weakened enamel (Fig. 5B). XRF analysis of gold-labeled enamel revealed differences in particle penetration and porosity distribution, with gold intensities normalized across particle sizes to enable direct comparison (Fig. 5D). The smallest particles (20 nm) showed a uniform gradient from the surface down to ~17 µm, with decreasing gold intensity at greater depths—closely mirroring the microhardness-affected zone (Fig. 5C, D). Larger particles (100 nm and 250 nm) exhibited reduced penetration, with increasingly patchy surface distributions. The 250-nm particles showed shallow penetration to around 6 µm, followed by a secondary increase in gold counts, possibly.

**Figure 5.**
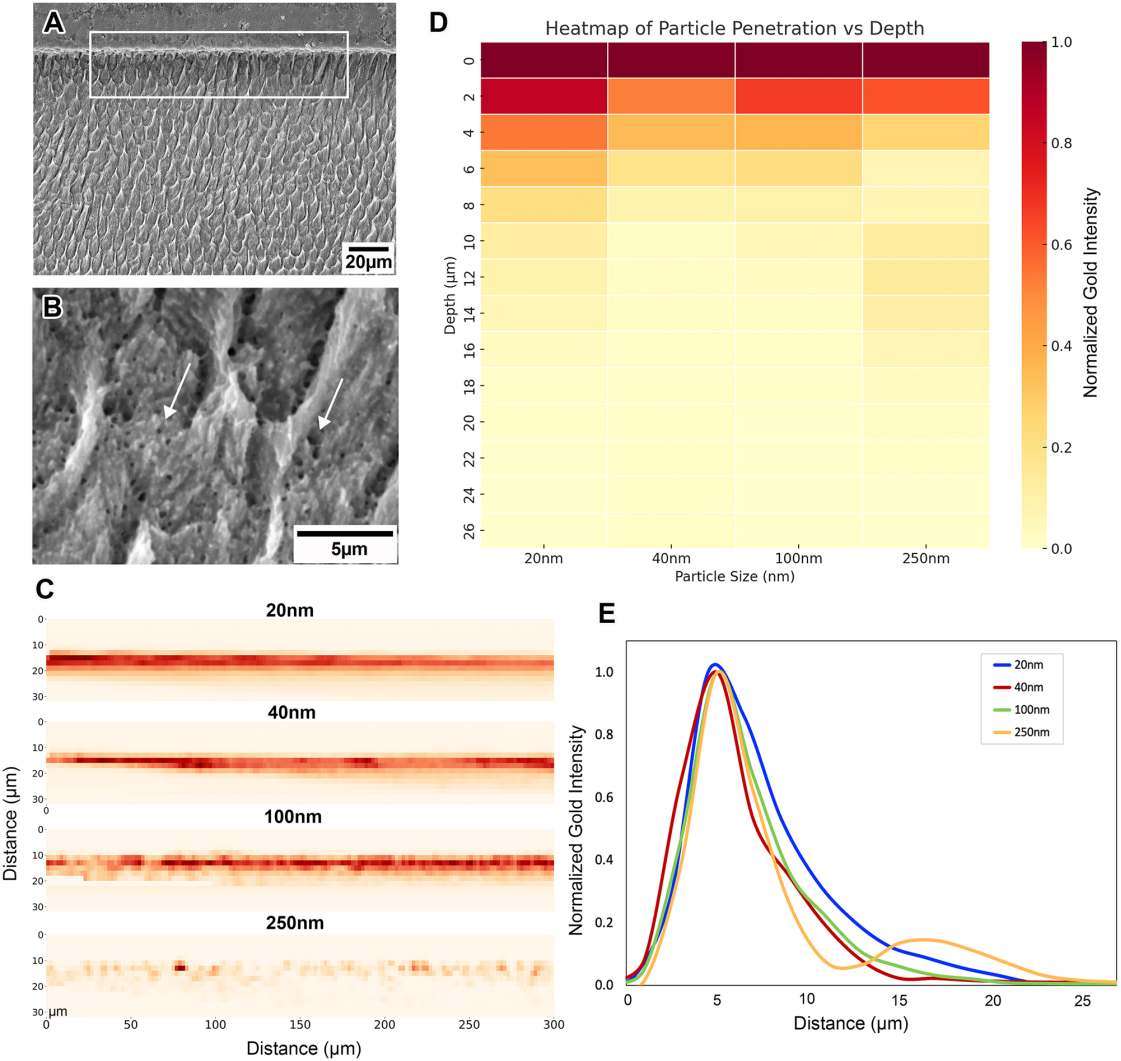
Synchrotron X-ray fluorescence (XRF) mapping of gold nanoparticle infiltration into enamel eroded for 10 min. (**A**) Overview image under scanning electron microscopy (SEM) showing a representative region mapped using synchrotron XRF. (**B**) Cross-sectional view of enamel eroded for 10 min, revealing the presence of porosities (white arrows) under SEM. (**C**) Horizontal XRF maps showing the distribution and relative concentration of gold for each nanoparticle size. Darker red areas indicate higher gold intensity. Each map is scaled independently and not normalized across particle sizes. (**D, E**) Vertical XRF maps and corresponding line graphs showing normalized gold intensity as a function of depth from the enamel surface. The 20-nm particles exhibit a uniform decrease with depth, similar to the 40-nm and 100-nm particles, although the latter show progressively shallower penetration. In contrast, the 250-nm particles display limited surface penetration with a mid-depth spike, indicating a more heterogeneous distribution pattern.

## Discussion

Using various indentation loads, we measured plastic deformation at different subsurface depths, allowing characterization of enamel’s mechanical response throughout the depth of the simulated erosive lesions over time. The response to indentation is known to depend on the enamel’s hierarchical microstructure and the direction and load of the indenter. Despite enamel’s brittle hydroxyapatite composition, it exhibits metallic-like behaviors (He and Swain 2007; Xie et al. 2009). Under light loads, the indenter interacts with enamel at the prismatic or interprismatic scale. During loading, interprismatic organic protein content permits elastic deformation (Guidoni et al. 2009), which recoils during unloading, reducing indent size and producing the indentation size effect, a phenomenon also observed by He and Swain (2007) with nanoindentation. This trend was clear in all control (noneroded) samples until a threshold load, beyond which hardness became load insensitive (Fig. 3G–I). Following citric acid exposure, particularly at early time points, this indentation size effect was lost, likely reflecting disruption of the organic matrix and decoupling of mineral phases.

As erosion progressed, light loads discriminated the mechanically weakest superficial layer, estimated at ~1.5-µm thick. Its fragility was further confirmed by ultrasonication, which removed the layer, increasing step height by 1.27 µm (±0.72) and 1.45 µm (±0.76) after 10- and 60-min exposures, respectively. Ultrasonication was previously used by Eisenburger et al. (2000), who characterized the entire subsurface thickness as being 2 to 5 µm. However, our results indicate that this value was an underestimation; what Eisenburger identified as the subsurface layer is only the softest, most superficial layer. Deeper layers, although partially demineralized, showed altered mechanical properties. Heavier indentation loads probed these zones, revealing higher hardness consistent with more intact enamel. Although deeper penetration (15–20 µm) was avoided to reduce the risk of crack, our results still demonstrated stratification and time-dependent changes in layer thickness. The removal of the outer layer, which is also likely to occur following toothbrushing, raises the question of whether it is salvageable via remineralization or if repair should focus on deeper, more stable regions.

We further evaluated time-dependent changes in the subsurface mechanical properties across a broad range of erosion times, from 20 s to 60 min. A sharp decline in hardness values was observed within the first minute when probed with light loads, likely reflecting early protein decoupling and initial weakening of the surface layer. Consistent with findings reported by Zheng et al (2009), enamel microhardness showed a significant decrease during the first 3 min before leveling off. However, the authors used only a single load of 0.05 kgF, whereas we observed a leveling off across 4 loads. While the longer erosion times of 10, 20, 40, and 60 min may not reflect typical clinical exposure, it was important for us to extend into these time frames to enable translation to other studies. Profilometry data reveal ongoing material loss, with step height increasing linearly over time, while the subsurface lesion depth remains stable. To our knowledge, this is the first study to demonstrate layer-specific changes showing that after approximately 3 min, the thickness of each subsurface layer remains unchanged despite continued erosion. This suggests a steady-state reaction, likely governed by the lesion’s exposed surface area and diffusion limitations—specifically, the inward movement of protons into the subsurface and the outward diffusion of dissolved calcium and phosphate ions (Cho et al. 2022).

Although this trend of increasing step height is consistent with prior literature, measuring surface loss during very short erosion times is challenging. Higher-than-expected step heights were recorded at 40 s and 1 min. Mylonas et al. (2019) noted that measurements of less than 60 s may be unreliable on polished surfaces. These anomalies could be due to surface reprecipitation at early stages or optical limitations during profilometry, such as refractive index changes or altered scattering on porous enamel.

Examining the network porosity and penetrability of the affected enamel surfaces is essential, particularly in hydrated samples, to ensure clinical relevance. Many existing techniques rely on thin sectioning, which risks surface damage and depends heavily on operator skill, limiting reproducibility. Our method avoids these issues, enabling direct quantification of pore size, distribution, and depth in minimally processed hydrated enamel. Our results indicate a compromised enamel structure, with widened prism sheaths (Appendix Fig. 2) and pore sizes of at least 20 nm extending to depths of ~17 µm. The more severely affected superficial layer exhibited patchy porosity of at least 250 nm, supporting previous findings that the uppermost 2 µm of enamel is the most vulnerable. However, penetrability decreased sharply beyond 6 µm, allowing only smaller particles to reach deeper regions. This method presents a promising approach for labeling porosities formed in hydrated samples after erosion using gold-coated nanoparticles, which offer greater contrast in XRF compared with air-filled voids or calcium, thereby improving detection and differentiation.

Although we cannot draw definitive conclusions on the effect of erosion on enamel porosity due to the limited sample size, the approach is highly reproducible, broadly adaptable, and minimally affected by sample preparation, supporting its use across diverse experimental settings. Incorporating nonembedding techniques, such as freeze drying, has been demonstrated to improve the preservation of fine surface features (Eisenburger et al. 2004). Although this study used Au L-edge fluorescence, excitation of the Au K-edge (80.7 keV) would permit analysis of thicker samples (>1 mm). Future work will extend these findings by combining porosity measurements with additional laboratory techniques and 3D synchrotron approaches, enabling smaller voxel sizes and full 3D visualization of gold distribution within enamel.

## Conclusion

This study reveals stratified mechanical properties and porosity in the subsurface layers of eroded enamel. Gold nanoparticle infiltration combined with synchrotron measurements enables high-resolution porosity mapping on hydrated, minimally prepared samples, providing a deeper understanding of lesion architecture and its link to mechanical integrity. These advances could support next-generation enamel care, shifting from bulk mineral delivery to layer-specific, functionally informed repair, including tailored particle sizes for targeted subsurface penetration.

## Supplemental Material

sj-docx-1-jdr-10.1177_00220345251385464 – Supplemental material for Beyond the Surface: Mechanical and Porosity Gradients in Eroded Enamel Supplemental material, sj-docx-1-jdr-10.1177_00220345251385464 for Beyond the Surface: Mechanical and Porosity Gradients in Eroded Enamel by H.L. Ooi, D. Bartlett, A. Almansour, A. LeBlanc, D. White, A. Morrell and O. Addison in Journal of Dental Research

Author Contributions

H.L. Ooi contributed to the conception and design of the study, data acquisition, analysis, and interpretation; drafted and critically revised the manuscript. D. Bartlett contributed to data interpretation and critically revised the manuscript. A. Almansour contributed to study design and critically revised the manuscript. A. LeBlanc contributed to data acquisition and critically revised the manuscript. Daniel White contributed to study design and critically revised the manuscript. A. Morrell contributed to data interpretation and critically revised the manuscript. O. Addison contributed to the conception and design of the study, data acquisition, analysis, and interpretation, and critically revised the manuscript. All authors gave final approval and agree to be accountable for all aspects of the work.
