# Supplementary material for: Beyond the Surface: Mechanical and Porosity Gradients in Eroded Enamel
Source: J Dent Res. 2025 Oct 22;105(7):883–91. doi: 10.1177/00220345251385464 (PMC13232576; doi:10.1177/00220345251385464)
Supplement: Supplementary file 1 [file sj-docx-1-jdr-10.1177_00220345251385464.docx]

Supplementary Material

**Beyond the Surface: Mechanical and Porosity Gradients in Eroded Enamel**

Hui Lynn Ooi^1^, David Bartlett^1^, Abdullah Almansour^1^, Aaron LeBlanc^1^, Daniel White^1^, Alexander Morrell^1,2^ & Owen Addison^1^

^1^ Faculty of Dentistry, Oral and Craniofacial Sciences, King’s College London, London, UK.

^2^ London Metallomics Facility, King’s College London, London, UK.

Corresponding Author: Hui Lynn Ooi

E-mail: [huilynn.ooi@kcl.ac.uk](mailto:huilynn.ooi@kcl.ac.uk)

Faculty of Dentistry, Oral and Craniofacial Sciences, King’s College London, London, UK.

**Supplementary Methods**

**Supplementary Figure 1:** Details on indentation geometry and indentation depth (h) calculation, with examples

of actual indent markings using ten different loads

Attenuation Coefficient

The attenuation depth was estimated based on the Beer-Lambert law, representing the depth at which X-ray intensity decreases to 37% of its initial value (1/e). Theoretical calculations were performed using the NIST X-ray Form Factor, Attenuation, and Scattering Tables, assuming hydroxyapatite (Ca₁₀P₆O₂₆H₂) as the target material, with an enamel density of 2.2–2.4 g/cm³ and the gold Lα emission energy of 9.65 keV. Although data acquisition was conducted in two dimensions 2µm x 2µm, the effective attenuation length (~90 µm) provides volumetric information, offering a quasi-3D representation of porosity and reducing artefacts associated with surface-only measurements.

Sample Size Calculation

A sample size calculation was conducted using G*Power version 3.1, based on pilot data comparing microhardness measurements across erosion times. With an effect size of 2.8, a significance level of α = 0.05, and power of 0.80, the minimum total sample size required was N = 6 (n = 2 per group). Our actual sample size of N = 24 (n = 8 per group) therefore exceeds the minimum required to test our hypothesis.

**Supplementary Table 1:** Parameters for Nanoparticles Used

| **Parameter** | **Value** |
| --- | --- |
| Core–Shell Composition | Au-coated Fe₃O₄ |
| Diameter (nm) | 20 ± 5 |
| Polydispersity Index (PDI) | 15% |
| Surface Charge (Zeta Potential, mV) | -15 |
| SPR Peak (nm) | 524 |
| Concentration (particles/ml) | 4.91 × 10^13^ |
| Solvent | 18 MEG deionized water |

| **Parameter** | **Value** |
| --- | --- |
| Core–Shell Composition | Au-coated Fe₃O₄ |
| Diameter (nm) | 40 ± 5 |
| Polydispersity Index (PDI) | 15% |
| Surface Charge (Zeta Potential, mV) | -21 |
| SPR Peak (nm) | 528 |
| Concentration (particles/ml) | 7.36 × 10¹² |
| Solvent | 18 MEG deionized water |

| **Parameter** | **Value** |
| --- | --- |
| Core–Shell Composition | Au-coated Fe₃O₄ |
| Diameter (nm) | 100 |
| Polydispersity Index (PDI) | 15% |
| Surface Charge (Zeta Potential, mV) | -15 |
| SPR Peak (nm) | 566 |
| Concentration (particles/ml) | 5.02 × 10^11^ |
| Solvent | 18 MEG deionized water |

| **Parameter** | **Value** |
| --- | --- |
| Core–Shell Composition | Au-coated Fe₃O₄ |
| Diameter (nm) | 250 |
| Polydispersity Index (PDI) | 15% |
| Surface Charge (Zeta Potential, mV) | -16 |
| SPR Peak (nm) | 538 |
| Concentration (particles/ml) | 2.71 × 10^10^ |
| Solvent | 18 MEG deionized water |

**Supplementary Table 2: Cohen’s d**

**Pre-Ultrasonication**

| Force (kg/F) | 1 min vs 10 min | 1 min vs 60 min | 10min vs 60 min |
| --- | --- | --- | --- |
| 0.01 | 5.90 | 5.50 | 0.92 |
| 0.02 | 6.40 | 5.36 | 0.13 |
| 0.025 | 3.85 | 5.28 | 1.04 |
| 0.05 | 3.57 | 5.25 | 0.51 |
| 0.1 | 4.10 | 2.89 | 0.74 |
| 0.2 | 3.19 | 3.21 | 1.12 |
| 0.3 | 2.99 | 2.91 | 0.71 |
| 0.5 | 1.77 | 2.48 | 1.20 |
| 1 | 1.70 | 1.24 | 0.21 |
| 2 | 1.11 | 0.55 | 0.46 |

**Post-Ultrasonication**

| Force (kg/F) | 1 min vs 10 min | 1 min vs 60 min | 10 min vs 60 min |
| --- | --- | --- | --- |
| 0.01 | 4.78 | 2.23 | 0.36 |
| 0.02 | 3.75 | 2.38 | 0.39 |
| 0.025 | 2.48 | 1.89 | 0.52 |
| 0.05 | 2.10 | 2.15 | 0.83 |
| 0.1 | 1.61 | 2.03 | 1.12 |
| 0.2 | 0.75 | 1.14 | 0.67 |
| 0.3 | 1.73 | 1.81 | 0.85 |
| 0.5 | 1.76 | 1.66 | 0.19 |
| 1 | 1.31 | 2.39 | 0.76 |
| 2 | 1.47 | 1.75 | 0.77 |

**Supplementary Table 3:** Data Tables

**Vicker’s Hardness Number**

| 60 mins | Pre-Ultrasonication | | Post-Ultrasonication | | Non-Eroded |  |
| --- | --- | --- | --- | --- | --- | --- |
| Force (kg/F) | Mean VHN (n=8) | Std | Mean VHN (n=8) | Std | Mean VHN (n=8) | Std |
| 0.01 | 135.93 | 31.28 | 205.40 | 87.35 | 442.81 | 51.74 |
| 0.02 | 191.59 | 30.20 | 247.79 | 64.64 | 441.04 | 32.79 |
| 0.025 | 192.28 | 25.78 | 240.24 | 82.41 | 427.83 | 37.84 |
| 0.05 | 228.19 | 21.34 | 273.53 | 55.01 | 416.64 | 27.31 |
| 0.1 | 250.55 | 43.34 | 288.68 | 39.69 | 386.78 | 29.61 |
| 0.2 | 265.57 | 33.46 | 311.85 | 33.01 | 382.29 | 55.10 |
| 0.3 | 282.15 | 30.30 | 307.76 | 39.02 | 353.43 | 52.14 |
| 0.5 | 291.70 | 18.57 | 318.92 | 22.17 | 333.02 | 50.16 |
| 1 | 295.00 | 17.95 | 283.84 | 12.86 | 317.81 | 56.42 |
| 2 | 309.40 | 19.69 | 289.72 | 23.43 | 307.33 | 43.93 |

**Indentation Depth**

| 60 mins | Pre-Ultrasonication | | Post-Ultrasonication | | Non-Eroded |  |
| --- | --- | --- | --- | --- | --- | --- |
| Force (kg/F) | Mean depth (µm) | Std | Mean depth (µm) | Std | Mean depth (µm) | Std |
| 0.01 | 1.63 | 0.16 | 1.43 | 0.27 | 0.93 | 0.05 |
| 0.02 | 1.92 | 0.15 | 1.79 | 0.23 | 1.32 | 0.05 |
| 0.025 | 2.20 | 0.21 | 2.06 | 0.36 | 1.49 | 0.07 |
| 0.05 | 2.78 | 0.29 | 2.66 | 0.24 | 2.13 | 0.07 |
| 0.1 | 3.76 | 0.33 | 3.64 | 0.25 | 3.13 | 0.12 |
| 0.2 | 5.15 | 0.36 | 4.95 | 0.29 | 4.48 | 0.35 |
| 0.3 | 6.22 | 0.56 | 6.11 | 0.41 | 5.71 | 0.49 |
| 0.5 | 7.83 | 0.61 | 7.71 | 0.27 | 7.60 | 0.62 |
| 1 | 10.95 | 1.38 | 11.55 | 0.26 | 11.04 | 1.13 |
| 2 | 15.12 | 1.90 | 16.20 | 0.66 | 15.80 | 1.19 |

**Vicker’s Hardness Number**

| 10 mins | Pre-Ultrasonication | | Post-Ultrasonication | | Non-Eroded |  |
| --- | --- | --- | --- | --- | --- | --- |
| Force (kg/F) | Mean VHN (n=8) | Std | Mean VHN (n=8) | Std | Mean VHN (n=8) | Std |
| 0.01 | 160.10 | 20.06 | 228.28 | 23.36 | 428.62 | 50.52 |
| 0.02 | 195.13 | 22.09 | 267.23 | 27.47 | 403.20 | 31.89 |
| 0.025 | 221.06 | 29.47 | 273.49 | 36.96 | 398.77 | 27.38 |
| 0.05 | 241.82 | 31.26 | 309.94 | 28.76 | 380.94 | 18.98 |
| 0.1 | 275.09 | 17.23 | 322.61 | 16.11 | 367.63 | 15.55 |
| 0.2 | 295.62 | 17.92 | 329.39 | 16.86 | 367.30 | 21.55 |
| 0.3 | 300.15 | 19.31 | 332.99 | 15.05 | 367.42 | 18.02 |
| 0.5 | 310.40 | 11.86 | 322.49 | 15.60 | 359.40 | 9.35 |
| 1 | 292.02 | 9.92 | 296.74 | 20.18 | 331.90 | 11.49 |
| 2 | 301.17 | 15.93 | 304.01 | 11.79 | 316.57 | 7.33 |

**Indentation Depth**

| 10 mins | Pre-Ultrasonication | | Post-Ultrasonication | | Non-Eroded |  |
| --- | --- | --- | --- | --- | --- | --- |
| Force (kg/F) | Mean Depth (µm) | Std | Mean Depth (µm) | Std | Mean Depth (µm) | Std |
| 0.01 | 1.54 | 0.10 | 1.29 | 0.07 | 0.94 | 0.06 |
| 0.02 | 1.98 | 0.11 | 1.69 | 0.08 | 1.37 | 0.05 |
| 0.025 | 2.08 | 0.15 | 1.87 | 0.12 | 1.54 | 0.05 |
| 0.05 | 2.81 | 0.20 | 2.48 | 0.11 | 2.23 | 0.06 |
| 0.1 | 3.71 | 0.11 | 3.43 | 0.08 | 3.21 | 0.07 |
| 0.2 | 5.06 | 0.15 | 4.80 | 0.12 | 4.54 | 0.13 |
| 0.3 | 6.15 | 0.20 | 5.84 | 0.13 | 5.56 | 0.13 |
| 0.5 | 7.81 | 0.15 | 7.66 | 0.18 | 7.25 | 0.09 |
| 1 | 11.38 | 0.19 | 11.30 | 0.40 | 10.68 | 0.19 |
| 2 | 15.86 | 0.41 | 15.78 | 0.32 | 15.45 | 0.18 |

**Vicker’s Hardness Number**

| 1 min | Pre-Ultrasonication | | Post-Ultrasonication | | Non-Eroded |  |
| --- | --- | --- | --- | --- | --- | --- |
| Force (kg/F) | Mean VHN (n=8) | Std | Mean VHN (n=8) | Std | Mean VHN (n=8) | Std |
| 0.01 | 286.01 | 22.55 | 349.90 | 27.35 | 412.75 | 19.24 |
| 0.02 | 326.40 | 18.78 | 363.55 | 23.83 | 407.53 | 21.57 |
| 0.025 | 326.49 | 25.06 | 357.55 | 30.63 | 391.69 | 17.94 |
| 0.05 | 333.95 | 18.89 | 364.06 | 22.52 | 383.87 | 12.57 |
| 0.1 | 346.16 | 17.43 | 355.35 | 23.91 | 371.20 | 18.76 |
| 0.2 | 349.37 | 15.68 | 348.10 | 30.80 | 370.77 | 24.09 |
| 0.3 | 351.67 | 14.93 | 364.48 | 20.83 | 363.45 | 6.74 |
| 0.5 | 338.66 | 19.23 | 353.92 | 19.83 | 347.21 | 23.30 |
| 1 | 318.14 | 19.36 | 322.19 | 18.73 | 311.45 | 16.35 |
| 2 | 319.42 | 16.88 | 325.12 | 16.55 | 309.58 | 8.84 |

**Indentation Depth**

| 1 min | Pre-Ultrasonication | | Post-Ultrasonication | | Non-Eroded |  |
| --- | --- | --- | --- | --- | --- | --- |
| Force (kg/F) | Mean Depth (µm) | Std | Mean Depth (µm) | Std | Mean Depth (µm) | Std |
| 0.01 | 1.15 | 0.04 | 1.04 | 0.04 | 0.96 | 0.02 |
| 0.02 | 1.52 | 0.04 | 1.44 | 0.05 | 1.36 | 0.04 |
| 0.025 | 1.71 | 0.06 | 1.63 | 0.07 | 1.55 | 0.04 |
| 0.05 | 2.38 | 0.07 | 2.28 | 0.07 | 2.22 | 0.04 |
| 0.1 | 3.31 | 0.08 | 3.27 | 0.11 | 3.19 | 0.08 |
| 0.2 | 4.66 | 0.10 | 4.67 | 0.22 | 4.52 | 0.16 |
| 0.3 | 5.68 | 0.12 | 5.58 | 0.16 | 5.59 | 0.05 |
| 0.5 | 7.48 | 0.21 | 7.32 | 0.21 | 7.39 | 0.27 |
| 1 | 10.91 | 0.33 | 10.84 | 0.32 | 11.03 | 0.30 |
| 2 | 15.40 | 0.40 | 15.26 | 0.39 | 15.63 | 0.23 |

**Step Height (Pre and Post Ultrasonication)**

| Erosion Time | Pre-Ultrasonication | | Post-Ultrasonication | |
| --- | --- | --- | --- | --- |
|  | Mean (µm) | Std | Mean (µm) | Std |
| 60 min | 16.24 | 2.99 | 17.69 | 2.77 |
| 10 min | 4.33 | 0.43 | 5.60 | 0.62 |
| 1 min | 5.61 | 1.07 | 4.21 | 0.92 |

**Vicker’s Hardness vs Erosion Time Repeated Measures**

| Load (kgF) | 0.01 |  | 0.02 |  | 0.1 |  | 0.5 |  |
| --- | --- | --- | --- | --- | --- | --- | --- | --- |
| Erosion Time (min) | Mean (VHN) | Std | Mean (VHN) | Std | Mean (VHN) | Std | Mean (VHN) | Std |
| 0 | 426.90 | 18.91 | 410.16 | 20.07 | 378.86 | 16.86 | 359.42 | 6.45 |
| 0.334 | 397.09 | 10.56 | 393.49 | 11.34 | 372.85 | 11.96 | 357.33 | 19.17 |
| 0.667 | 329.47 | 21.07 | 350.85 | 13.69 | 355.50 | 16.79 | 348.00 | 11.23 |
| 1 | 293.71 | 15.90 | 313.84 | 17.40 | 343.49 | 22.48 | 342.70 | 11.18 |
| 2 | 242.89 | 14.07 | 277.05 | 15.68 | 318.77 | 23.09 | 334.98 | 13.70 |
| 3 | 229.30 | 21.97 | 261.39 | 32.04 | 308.88 | 12.71 | 330.32 | 14.40 |
| 4 | 231.93 | 27.53 | 274.20 | 24.41 | 310.02 | 6.63 | 335.30 | 10.95 |
| 5 | 225.56 | 17.26 | 260.95 | 15.00 | 313.98 | 8.42 | 335.49 | 12.78 |
| 10 | 201.14 | 23.21 | 251.31 | 22.92 | 301.50 | 22.33 | 335.99 | 14.52 |
| 20 | 174.45 | 26.07 | 242.31 | 34.63 | 302.98 | 27.83 | 331.08 | 17.47 |
| 40 | 146.17 | 38.18 | 182.32 | 31.17 | 278.73 | 34.05 | 329.71 | 12.82 |
| 60 | 181.71 | 63.16 | 204.48 | 44.60 | 292.29 | 21.78 | 328.51 | 16.09 |

**Step Height vs Erosion Time**

| Time (min) | Mean Step Height (µm) | Std |
| --- | --- | --- |
| 0.334 | 2.28 | 0.83 |
| 0.667 | 5.19 | 1.29 |
| 1 | 5.45 | 1.21 |
| 2 | 3.65 | 1.87 |
| 3 | 2.62 | 1.10 |
| 4 | 2.76 | 0.89 |
| 5 | 2.76 | 0.81 |
| 10 | 3.63 | 0.83 |
| 20 | 6.37 | 1.03 |
| 40 | 11.47 | 1.29 |
| 60 | 17.57 | 1.60 |

**Supplementary Figure 2:** Optical microscope images of the facial surface of (A) eroded and (B) uneroded enamel surface after infiltration with gold magnetic nanoparticles. (A) shows the nanoparticles infiltrating the widened prism sheaths.
